# Quantification of pathogenic *Leptospira* in the soils of a Brazilian urban slum

**DOI:** 10.1371/journal.pntd.0006415

**Published:** 2018-04-06

**Authors:** Andrew G. Schneider, Arnau Casanovas-Massana, Kathryn P. Hacker, Elsio A. Wunder, Mike Begon, Mitermayer G. Reis, James E. Childs, Federico Costa, Janet C. Lindow, Albert I. Ko

**Affiliations:** 1 Department of Epidemiology of Microbial Diseases, School of Public Health, Yale University, New Haven, Connecticut, United States of America; 2 Centro de Pesquisas Gonçalo Moniz, Fundação Oswaldo Cruz, Ministério da Saúde, Salvador, Bahia, Brazil; 3 Institute of Integrative Biology, University of Liverpool, Liverpool, United Kingdom; 4 Instituto de Saúde Coletiva, Universidade Federal da Bahia, Salvador, Bahia, Brazil; University of Connecticut Health Center, UNITED STATES

## Abstract

**Background:**

Leptospirosis is an important zoonotic disease that causes considerable morbidity and mortality globally, primarily in residents of urban slums. While contact with contaminated water plays a critical role in the transmission of leptospirosis, little is known about the distribution and abundance of pathogenic *Leptospira* spp. in soil and the potential contribution of this source to human infection.

**Methods/Principal findings:**

We collected soil samples (n = 70) from three sites within an urban slum community endemic for leptospirosis in Salvador, Brazil. Using qPCR of *Leptospira* genes *lipl32* and 16S rRNA, we quantified the pathogenic *Leptospira* load in each soil sample. *lipl32* qPCR detected pathogenic *Leptospira* in 22 (31%) of 70 samples, though the median concentration among positive samples was low (median = 6 GEq/g; range: 4–4.31×10^2^ GEq/g). We also observed heterogeneity in the distribution of pathogenic *Leptospira* at the fine spatial scale. However, when using 16S rRNA qPCR, we detected a higher proportion of *Leptospira*-positive samples (86%) and higher bacterial concentrations (median: 4.16×10^2^ GEq/g; range: 4–2.58×10^4^ GEq/g). Sequencing of the qPCR amplicons and qPCR analysis with all type *Leptospira* species revealed that the 16S rRNA qPCR detected not only pathogenic *Leptospira* but also intermediate species, although both methods excluded saprophytic *Leptospira*. No significant associations were identified between the presence of pathogenic *Leptospira* DNA and environmental characteristics (vegetation, rat activity, distance to an open sewer or a house, or soil clay content), though samples with higher soil moisture content showed higher prevalences.

**Conclusion/Significance:**

This is the first study to successfully quantify the burden of pathogenic *Leptospira* in soil from an endemic region. Our results support the hypothesis that soil may be an under-recognized environmental reservoir contributing to transmission of pathogenic *Leptospira* in urban slums. Consequently, the role of soil should be considered when planning interventions aimed to reduce the burden of leptospirosis in these communities.

## Author summary

Leptospirosis is a globally distributed zoonotic disease that disproportionately affects vulnerable populations in urban slums. The disease is transmitted by direct contact with water, soil, or mud that has been contaminated with infected urine shed from chronically infected animals. Despite the critical role the environment plays in the epidemiology of the disease, the contribution of soil to the transmission cycle remains largely undescribed. Herein, we investigated the distribution of pathogenic *Leptospira* in soil samples from an endemic urban slum in Brazil. We found pathogenic *Leptospira* in nearly one-third of the soil samples, predominantly in low concentrations (<5×10^2^ GEq/g). However, we observed considerable variation in the distribution and concentration of the pathogen at the fine spatial scale within the slum. Our results indicate that soil is likely an important additional environmental reservoir of pathogenic *Leptospira* in urban slums and prevention strategies should consider soil to help prevent the transmission of the disease in similar settings.

## Introduction

Leptospirosis is a life-threatening, zoonotic disease of global importance, with more than 1 million cases and approximately 60,000 deaths estimated annually, predominately in developing tropical countries [1]. The disease is caused by spirochetes of the genus *Leptospira*, which contains 35 species, 13 of which in the pathogenic group [2,3]. Pathogenic *Leptospira* chronically colonize the renal tubules of animal reservoirs and are excreted with the urine. Humans are incidental hosts, and often acquire the infection after seasonal or intense precipitation events [4–6], when pathogenic *Leptospira* in contaminated soil, mud, or water penetrate abraded skin or wounds [7,8]. Clinical manifestations of leptospirosis range from mild or asymptomatic to severe illness, such as Weil’s disease [7,9], and pulmonary hemorrhage syndrome [9,10], which cause high case fatality rates.

Urban leptospirosis has emerged as a pandemic which disproportionately affects residents of slum communities around the world [11]. Poor sanitation and an abundance of food sources provide ideal conditions for the maintenance of large rodent populations, specifically the Norway rat (*Rattus norvegicus*), which are the primary animal reservoirs of pathogenic *Leptospira* in urban environments [12–16]. Infrastructure deficiencies facilitate the transmission to humans [17–19]: Open sewers and inadequate drainage systems allow contaminated water to pervade surrounding soil and water, and unpaved walkways expose residents to contaminated dirt and mud. Thus, the human-environment interface plays a critical role in the epidemiology and transmission of leptospirosis in urban slums. While previous studies have found *Leptospira* spp. in puddles, sewers, streams, and soil in endemic regions [20–25], the distribution and dynamics of leptospires in the environment, particularly in soil, are largely unknown.

To date, few publications have reported pathogenic *Leptospira* in soil, presumably because leptospirosis is generally considered to be a water-borne disease, and thus environmental studies have focused preferentially on aquatic matrices [20,21]. Previous studies that successfully analyzed soil reported very low prevalence among samples (4.9% in China [26]), or the isolation of only a few pathogenic strains in Philippines, Malaysia and New Caledonia [2,23,24,27,28]. These studies, however, were based on culture isolation of leptospires, followed by molecular identification. Culture techniques lack sensitivity because of pathogenic *Leptospira* spp. being overgrown by the autochthonous soil microbiota or saprophytic and intermediate *Leptospira* [23,24,29]. Notably, a recent study by Thibeaux et al. [25] reported a 57.7% prevalence in soil samples from a suspected environmental infection site in New Caledonia by using a qPCR targeting *lipL32*.

In this study, we aimed to quantify the pathogenic *Leptospira* load in soil samples from an urban slum community in Salvador, Brazil. Previous studies have shown this community has a high burden of disease [6,18,30] and a widespread presence of pathogenic *Leptospira* in its surface waters [31], making it an excellent location for an environmental survey of the pathogen. Since Norway rats are the predominant pathogenic *Leptospira* reservoir in this community [14], we hypothesized that the presence of rats would be associated with the occurrence and abundance of the pathogen in soil. As urban slum communities in tropical regions share many socioeconomic, structural, and environmental characteristics [32,33], our study may help inform potential public health interventions in similar epidemiological settings around the world.

## Methods

### Study site

**Fig 1:**
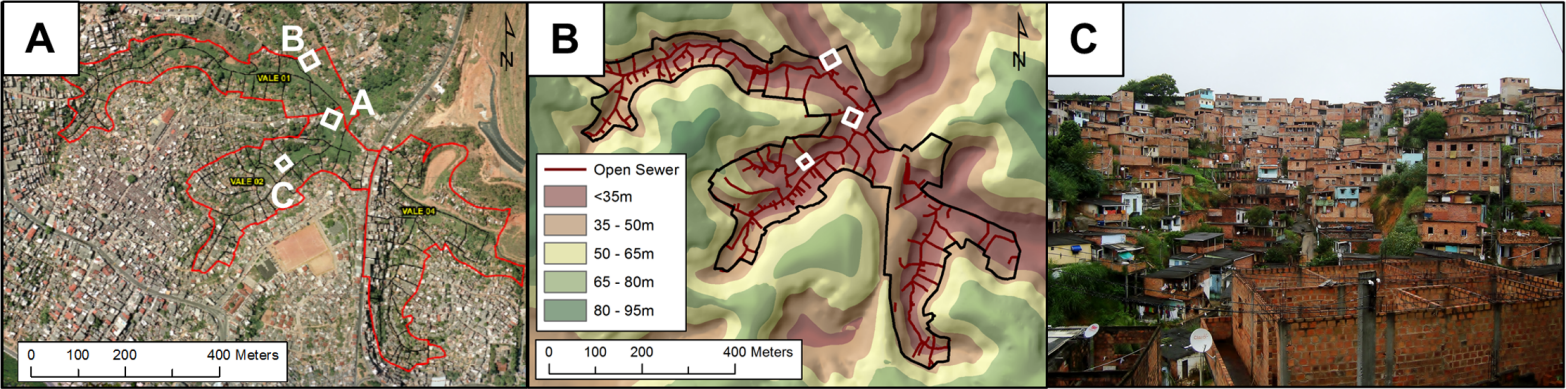
The Pau da Lima community site in Salvador, Brazil. (A) Aerial photograph of the community site (red border), which encompasses three valleys. The locations of the three sites of environmental sampling are indicated by white rectangles. (B) Topographic map that demonstrates differences in elevation at the site. Open sewage draining systems are depicted in dark red. (C) Photograph of the community, depicting a representative valley and squatter households. Panels A and B were created with ArcGIS using copyright-free aerial photographs provided by the Salvador municipality.

**Fig 2:**
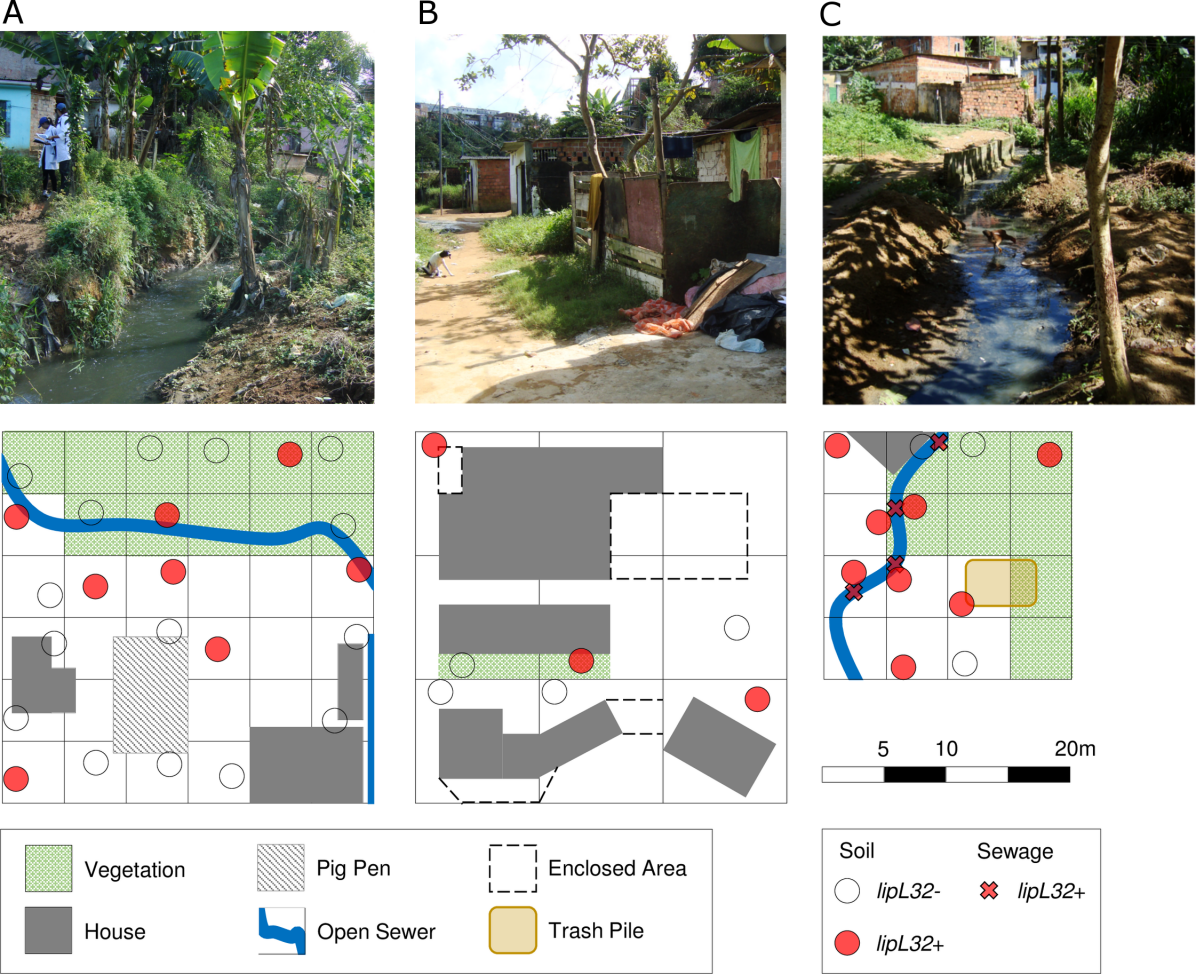
Environmental sampling and qPCR detection of *Leptospira* DNA in the community site. Sampling of soil and sewage (circles) at three sites (A, B, and C) within the Pau da Lima field site. Positive and negative samples by *lipL*32 qPCR are depicted as red and open circles, respectively.

We conducted this study in the community of Pau da Lima, an urban slum located in Salvador, Brazil. This site has been extensively described in previous studies [18,19]. Briefly, the community encompasses four interconnected valleys within 0.46 km^2^ (Fig 1A), with a population of 14,122 inhabitants residing in 3,689 households, according to a 2003 census [18]. The community lacks adequate sanitary infrastructure, resulting in untreated wastewater and rain runoff flowing through open sewers in the lower parts of the valleys. Leptospirosis is endemic in Pau da Lima, with a mean annual infection incidence of 37.8 per 1,000 inhabitants, and 19.8 severe cases per 100,000 inhabitants [30]. We selected three collection sites to represent a range of microenvironments present within the community (Fig 1B). Site A (900 m^2^) (12°55'22.2"S, 38°26'04.2"W) was located along an open sewer at the bottom of the valley, and included households, areas of domestic animal raising, and thick vegetation (Fig 2A). The steep, high banks of the sewer served as a barrier to separate households from the sewage, which limited potential flooding. Site B (900 m^2^) (12°55'17.9"S, 38°26'07.2"W) was situated at a higher elevation next to the valley slope and had closed sewage drains, paved stairs, and patios (Fig 2B). There was limited vegetation, although fences and barriers, coupled with the sheer valley slope, restricted access. Site C (400 m^2^) (12°55'24.8"S, 38°26'06.3"W) was situated at the bottom of the valley and in proximity to an open sewer with low embankments, allowing frequent flooding of surrounding areas during heavy rainfall periods. The thick vegetation and water-logged soil made part of the site inaccessible. We partitioned collection sites into 5 m x 5 m squares (A and C) or 10 m x 10 m (B) (Fig 2A and 2B). A larger grid was used at Site B, as many areas were impassible due to the decreased number of sampling locations and the challenging terrain (Fig 2B).

### Sample collection and rat activity monitoring

We collected soil and sewage samples in the rainy season between July and August 2014 (S1 Fig). A tracking board to monitor rat activity in the collection sites was placed as described previously [34] within all grid squares that were accessible and contained exposed soil. Tracking boards were evaluated daily over the course of three days for evidence of rat activity as ascertained by the identification of footprints, scrapes, and tail slides [34]. After the three days, one or two soil samples of 100–200 g were collected at a depth of 5–10 cm from non-adjacent areas within 30 cm of the edge of each tracking board between 9am and 12.30pm. Grid squares that were inaccessible because of private property, dense vegetation or water-logged soil, or contained no exposed soil were not included in the sampling. In total, we collected soil samples from 23, 7 and 11 grid squares in sites A, B and C, respectively for a total of 35, 14 and 22 soil samples. If a portion of an open sewer was included in the grid square and was accessible, two 50 mL samples for each sampling point were collected. The presence of vegetation and distance to open sewers and households was recorded for each grid square. The soil moisture and clay content were measured for one sample from each grid square. Samples were placed in aseptic containers, transported to the laboratory, and processed within 6 h.

### DNA extraction

To maximize the recovery of *Leptospira* DNA from soil samples, we developed an extraction protocol and determined its efficiency by performing spiking experiments with known concentrations of *L*. *interrogans* (S1 Supplementary methods). The final procedure consisted in the following steps: Subsamples of 5 g of soil were mixed with 40 mL of sterile double-distilled water and vortexed at maximum speed for 2 min. Samples were centrifuged at 100 rcf for 5 min. The supernatant was recovered and centrifuged at 12,000 rcf for 20 min at room temperature. The pellets were recovered, resuspended in 1.5 mL of sterile double-distilled water and centrifuged at 12,000 rcf for 20 min. Finally, the samples were decanted and the pellets were frozen at -80°C. Sewage samples (40 mL) were processed as described previously [35]. DNA was extracted from pellets using PowerSoil DNA Isolation Kit (Mo Bio Laboratories). An extraction blank (sterile double-distilled water) was added to each extraction batch to monitor for cross-contamination.

### qPCR

We quantified *Leptospira* spp. loads using two TaqMan assays targeting the *lipL32* gene or the 16S rRNA gene as described previously [36,37] with minor modifications. Briefly, all reaction mixtures (25 μL) contained 12.5 μL Platinum qPCR SuperMix (Life Technologies), 0.2 μg/μL of bovine serum albumin (Ambion), and 5 μL of DNA template. The *lipL32* reactions included 500 nM each of primers LipL32-45F and LipL32-286R, and 100 nM of LipL32-189P probe (S1 Table). 16S rRNA reactions included 300 nM each of primers Lepto F and Lepto R, and 200 nM of 16S probe. Amplifications were performed using a 7500 Fast Real-Time PCR System (Life Technologies) with the following conditions: an initial step of 2 min at 50°C, followed by 2 min at 95°C, and 40 cycles of amplification (15 s at 95°C and 1 min at 60°C). Genomic DNA obtained from *L*. *interrogans* serovar Fiocruz L1-130 [38] was used to construct calibration curves with concentrations ranging from 2 × 10^2^ to 2 × 10^9^ GEq/mL, which we included in each qPCR run. Efficiencies were always higher than 92.5%. All samples were run in duplicate, and included non-template controls in each plate row to detect any contaminating DNA. All negative controls (extraction controls and non-template controls) were negative. All DNA extractions and quantitative PCR (qPCR) analyses were performed according to the minimum information for publication of quantitative real-time PCR experiments (MIQE) guidelines [39].

To determine the specificity of the *lipL32* and 16S rRNA qPCRs for pathogenic, intermediate and saprophytic *Leptospira*, we tested DNA extracts from 21 *Leptospira* species (S2 Table) as explained above, adjusting the concentration in each well to 10^7^ GEq based on each genome’s size [40].

### Sequencing of *Leptospira* qPCR amplicons

To confirm the specificity of the environmental qPCR reactions, we randomly selected and sequenced (Sanger method) 11/22 (50%) of the soil samples with positive results for *lipl32*. We also sequenced 27/78 (35%) of samples with a positive result for 16S rRNA qPCR (25 soil and 2 sewage samples). In brief, qPCR products were purified from 2% agarose gels using the QIAquick Gel Extraction Kit (QIAgen) following the manufacturer’s instructions. The products were sequenced using primers LipL32-45F or Lepto-F for *lipL32* and 16S rRNA, respectively, corrected using BioEdit 7.2.0 (Ibis Biosciences), and the sequences compared using BLAST to those available in the NCBI. In addition, we performed a phylogenetic analysis for the *lipl32* and 16S rRNA amplicons using the Phylogeny.fr platform [41]. Maximum Likelihood trees were inferred by PhyML 3.0 [42] using the Hasegawa-Kishono-Yano (HKY85) substitution model and 1000 bootstrap replicates. We used FigTree v1.4.2 (http://tree.bio.ed.ac.uk/software/figtree) to visualize and edit the final trees. The 16S rRNA tree included *Leptonema illini* DSM 21528 as the outgroup.

### Data treatment and statistical analyses

*Leptospira* concentrations were log_10_ transformed and analyzed as follows. The theoretical lower limit of quantification (LOQ) of the qPCR assays was calculated using the assumption that 1 copy of the targeted gene was amplified in a reaction (4 GEq/g). We assigned positive samples with concentrations below this threshold a value equal to the LOQ. Fisher’s exact test and the χ^2^ test were used to compare prevalence among sites and the associations of dichotomized environmental characteristics with the proportion of qPCR-positive samples. *t*-tests with a Welch’s correction were used to compare soil moisture content and clay component values between positive and negative qPCR samples. The median concentrations between sampling sites were compared using Kruskal-Wallis test with Dunn’s correction for multiple comparisons. Mann-Whitney test was used to compare the concentrations between soil and sewage at site C and the overall concentrations obtained by *lipl32* and 16S rRNA qPCRs. Non-parametric tests were chosen due to the non-normal distribution of the data. Although equivalent parametric tests (one-way ANOVA and t-test) were inappropriate, technically, they supported the results of the non-parametric tests in all cases. To analyze the degree of agreement between *lipL32* and 16S PCR detection methods, we used Cohen’s kappa coefficient. All statistical analyses were performed using GraphPad Prism 6.05 (GraphPad Software Inc.), and multivariate models were fit using the GENMOD procedure with a GEE model in SAS v9.3 (SAS Institute Inc., Cary, NC) to evaluate the relationship between the measured environmental characteristics and outcome of sample testing.

## Results and discussion

### Pathogenic *Leptospira* are present in soil using *lipL32* qPCR

**Fig 3:**
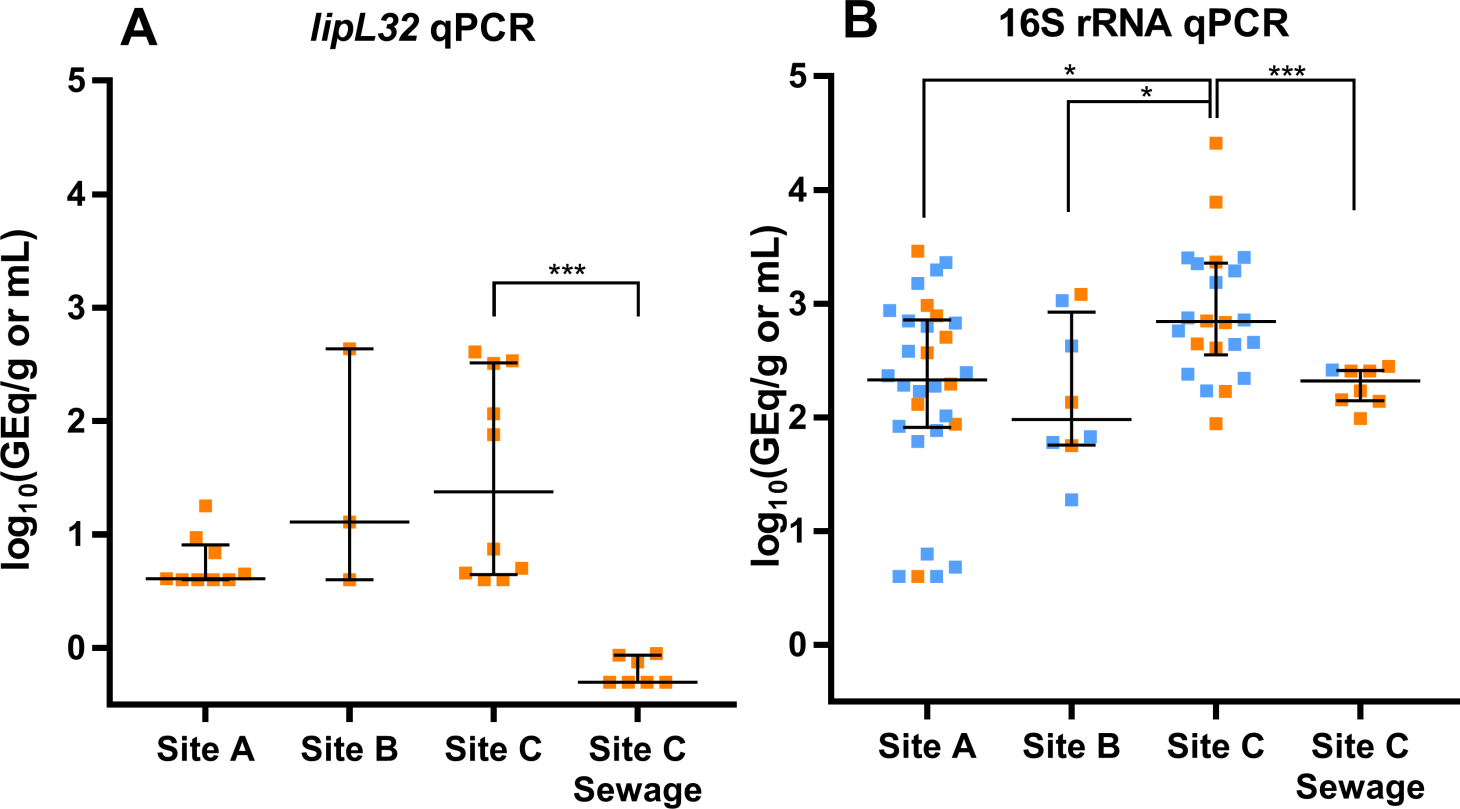
Pathogenic *Leptospira* concentrations in environmental samples. The median concentration and interquartile range is shown for each site. (A) Concentrations measured with *lipL32* qPCR. (B) Concentrations measured with 16S rRNA qPCR. Samples in orange were positive for both assays, while those in blue were only positive for the 16S rRNA qPCR. The differences between the soil median concentrations in the three sites were analyzed by Kruskal-Wallis test with Dunn’s correction for multiple comparisons. The differences between the median concentrations from soil and sewage at site C were analyzed with the Mann-Whitney test. (*) p≤0.05; (***) p≤0.0001.

We collected 70 soil samples within the study area (34, 14, and 22 from sites A, B, and C, respectively) (Fig 2). Of those 70 samples, 22 (31%) were positive for pathogenic *Leptospira* as measured by the *lipL32* qPCR (Fig 3A). There were no significant differences in the proportion of positive samples between the three collection sites (26%, 21% and 45%, respectively; p = 0.78) (Table 1), indicating that pathogenic *Leptospira* were widespread in the study site. These prevalences are lower than those recently reported using the same qPCR method in river bank soils from active leptospirosis transmission sites in New Caledonia (57.7%) [25]. Nevertheless, our results contrast with the very low number of pathogenic *Leptospira* isolates that are usually obtained from soil in endemic areas using culture-based approaches [23,24,26–28], suggesting that molecular approaches may capture better the presence of the pathogen in soil.

**Table 1:** Detection of pathogenic *Leptospira* by *lipL32* qPCR in soil samples according to environmental characteristics of the sampling sites within the urban slum community. None of the measured variables showed significant differences between *lipL32-*positive and *lipL32-*negative samples in bivariate or multivariate analysis.

		Soil Samples (n = 70) No. (%) or Mean (SD)	
Characteristic		*lipL32* Positive	*lipL32* Negative
**Site^a^**	**All**	22 (31%)	48
	**A**	9 (26%)	25
	**B**	3 (21%)	11
	**C**	10 (45%)	12
**Adjacent Vegetation**		14 (64%)	27 (56%)
**Evidence of Rat Activity**		16 (73%)	28 (58%)
**Distance to Open Sewer^b^**		3.3 (4.6)	5.3 (5.6)
**Distance to House^c^**		6.8 (4.5)	5.9 (5.7)
**Soil Moisture Content^d^**		28.4 (15.7)	26.8 (13.7)
**Soil Clay Content^d^^,^^e^**		10.7 (4.2)	10.8 (5.5)

^a^ Percent for the site variables are row percent. The others are column percent.

^b^ n = 56 at sites A and C combined. Site B did not have an open sewer. Measurements are in meters.

^c^ Measured in meters.

^d^ Percent moisture.

^e^ n = 56

The concentration of pathogenic *Leptospira* among positive samples was predominantly low (median: 6 GEq/g), but varied by two orders of magnitude (range: 4–4.31×10^2^ GEq/g) even within the same collection grid. This indicates a considerable heterogeneity of environmental loads within the slum microenvironment. Furthermore, there were no significant differences in the concentration of the qPCR-positive soil samples among the three collection sites (p = 0.16) (Fig 3A). Among the eight sewage-water samples collected from site C, seven (88%) were positive by the *lipL32* qPCR with a median concentration of 0.5 GEq/mL.

Together our results showed that pathogenic *Leptospira* were present in low concentrations in soils sampled from diverse microenvironments within the urban slum. Contact with mud in the peridomiciliary environment was previously identified as a risk factor for leptospirosis infection in this setting [18], which suggests that soil may serve as an important environmental reservoir of the pathogen. Intense rainfall events during the rainy season would cause mobilization and dispersion of pathogenic *Leptospira* from soil to run-off as described for other environmental pathogens such as *E*. *coli*, *Salmonella* spp., *Cryptosporidium* spp. and fecal indicator bacteria [43–48]. Simultaneously, run-off originated in the higher areas of the valley may contaminate soil in the lower areas with pathogenic *Leptospira* through flooding and sewer overflow. Thus, soil may act as a source and a recipient of the pathogen depending on the specific location and weather conditions. Furthermore, the low concentrations found in soil are in agreement with those found in sewage and standing water in a previous study conducted in the same setting [31], which supports the hypothesis that the environmental load of pathogenic *Leptospira* is generally low, even in endemic areas. The dynamics and characteristics of water-based mobilization and dispersion of *Leptospira* to and from the soil reservoir within the slum community, and the role that low environmental concentrations may have on the risk of acquiring leptospirosis, will require detailed studies beyond the scope of our methods.

### More soil samples contain *Leptospira* when using 16S rRNA qPCR

In contrast to the *lipL32* qPCR results, the 16S rRNA qPCR detected *Leptospira* from 60/70 (86%) soil samples, significantly more than detected by the *lipL32* qPCR (p<0.0001). Higher prevalences were found at sites A and C (88% and 100%, respectively) than site B (57%) (p<0.0014) (Table 2). Among positive samples, the median concentration was 4.16×10^2^ GEq/g (range: 4–2.58×10^4^ GEq/g), significantly higher than the one detected by *lipL32* qPCR (6 GEq/g, p < 0.0001). All eight sewage samples from Site C were also positive using the 16S qPCR, with a mean concentration of 2.09×10^2^ GEq/mL (range: 98–2.81×10^2^), nearly 9-fold higher than that detected by *lipL32* qPCR (24 GEq/mL, p = 0.0003). Notably, all soil and sewage samples that were positive using the *lipL32* assay were positive with the 16S assay, though there was a poor agreement between the qualitative results obtained by the two methods (Cohen’s Kappa coefficient = 0.14± 0.05) (Table 2)

**Table 2:** Summary of sampling data and results obtained by *lipl32* and 16S rRNA qPCR (presence/absence and concentration) for the three sites investigated.

			*lipl32*		16S rRNA		
	Site	N of samples	Positive samples	Concentration^a^	Positive samples	Concentration^a^	Agreement^b^
**Soil**	A	34	9 (26%)	4 (4–17.8)	30 (88%)	2.13×10^2^ (4–2.91×10^3^)	0.09 ± 0.05 Poor
	B	14	3 (21%)	13 (4–4.31×10^2^)	8 (57%)	96 (19–1.20×10^3^)	0.34 ± 0.18 Fair
	C	22	10 (45%)	24 (4–4.09×10^2^)	22 (100%)	6.95×10^2^ (88.5–2.57×10^4^)	0.00 ± 0.00 Poor
	All	70	22 (31%)	6 (4–4.31×10^2^)	60 (86%)	4.16×10^2^ (4–2.57×10^4^)	0.14 ± 0.05 Poor
**Sewage**	C	8	7 (88%)	0.5 (0.5–0.9)	8 (100%)	2.09×10^2^ (97.7–2.81×10^2^)	0.00 ± 0.00 Poor

^a^Median concentration and range (GEq/g or GEq/mL)

^b^Cohen’s kappa coefficient between qualitative *lipl32* and 16S rRNA qPCR results, standard error and strength of agreement.

### The 16S rRNA PCR method also detects intermediate group *Leptospira*

**Fig 4:**
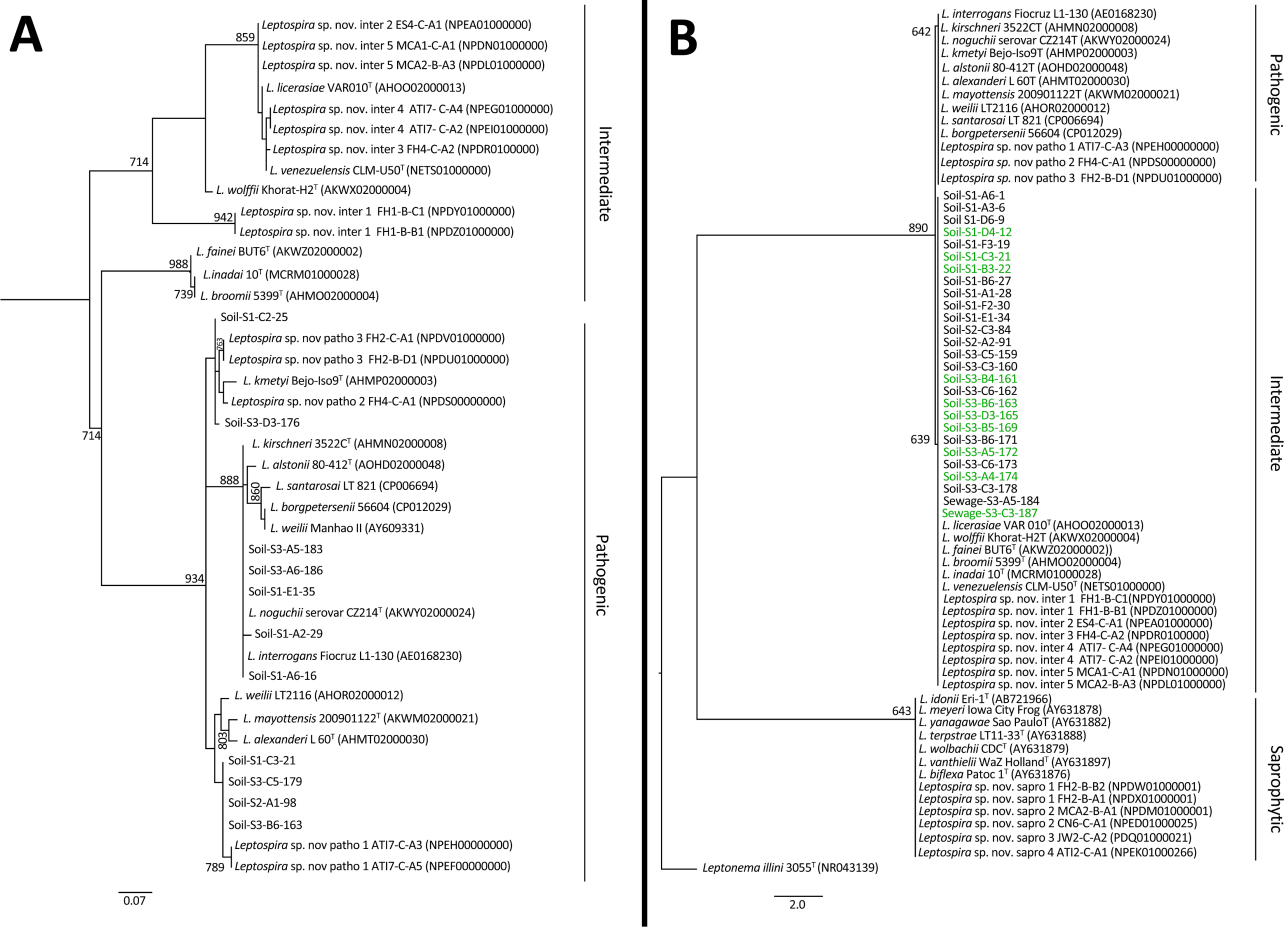
Phylogenetic trees of (A) *lipl32* and (B) 16S rRNA *Leptospira* amplicons from soil and water samples collected at the community site. The trees were constructed using Maximum Likelihood method with HKY85 substitution model. A bootstrap of 1000 replicates was performed and values above 600 are shown in the nodes. *Leptonema illini* was used as the outgroup for the 16S rRNA tree, and samples colored in green were positive for both 16S rRNA and *lipl32* qPCRs.

Given the discrepancy of results obtained with the two-qPCR methods (Table 2), we analyzed the specificity for detecting pathogenic *Leptospira* for each method. We sequenced the *lipL32* amplicon from 11/22 (50%) *lipL32* qPCR-positive soil samples. In all cases, the sequences presented similarities greater than 92% with *lipL32* gene sequences from pathogenic *Leptospira* deposited in GenBank (S3 Table) and clustered with species from the pathogenic group (Fig 4A). These results confirmed that the *lipL32* qPCR was highly specific for pathogenic *Leptospira* in soil as it was previously shown in sewage [31]. We also sequenced 27/78 (35%) 16S qPCR-positive samples (25 soil and 2 sewage samples). All the sequenced 16S rRNA amplicons clustered with species from the intermediate *Leptospira* group (Fig 4B). As observed elsewhere [49], a single mismatch in the approximately 50 bp fragment sequenced discriminates between intermediate and pathogenic *Leptospira* groups. Indeed, the reverse primer used in the 16S qPCR is degenerated at position 14 allowing for the hybridization with T and C bases, and thus, may detect both pathogenic and intermediate species (S1 Table). Of note, 6 sequences that were positive for both *lipL32* and 16S presented a double peak in the sequencing chromatogram at the mismatch position. This indicates that both sequences coexisted in the sample [49], although in all cases the highest peak was the one belonging to the intermediate group.

To conclusively determine the specificity of the both qPCRs, we tested 21 type strains from the pathogenic, intermediate and saprophytic groups. *Lipl32* qPCR showed signal only for pathogenic species, and excluded intermediate and saprophytic ones. In contrast, all pathogenic and intermediate species gave positive results for 16S rRNA qPCR and no signal was observed for the saprophytes (S2 Table). Therefore, 16S rRNA qPCR detects not only pathogenic *Leptospira*, but also intermediate species. Since the pathogenicity of the intermediate group is not well established, we considered only the results obtained with *lipL32* qPCR for subsequent analyses.

### The presence of *Leptospira* is not associated with specific environmental factors except moisture

Bivariate and multivariate analyses identified no significant associations between the presence of pathogenic *Leptospira* DNA in soil and environmental characteristics such as vegetation, distance to an open sewer or a house, or soil clay content (Table 1). However, we found that 62% of samples with a moisture content higher than 20% were positive, while only 21% were positive when the moisture was lower than 20%, which is consistent with previous observations that higher soil moisture content is associated with increased *Leptospira* isolation [23,50]. Additionally, previous studies have reported that *Leptospira* persist for a longer time in moist and super-saturated soils than in drier ones, although the duration of survival is also dependent on the serovar and other characteristics of the soil such as pH [51–53].

Furthermore, against our initial hypothesis, we did not find any significant association between rat activity and the presence of pathogenic *Leptospira* in soil. A potential explanation is that the direct association with rats is confounded by other animal sources of pathogenic *Leptospira* both domestic (cows, pigs and dogs) and wildlife (opossums and bats) that coexist in this urban slum [15,34]. Alternatively, as discussed above, some of the pathogenic *Leptospira* detected may have originated in other parts of the valley and contaminated the soil through run-off or floodwater, making rat activity an unreliable proxy for the presence of the pathogen. Finally, we cannot rule out that the tracking board method was not a sufficient to assess rat activity at the fine scale at which the variation of the presence and concentration of pathogenic *Leptospira* in the soil seems to occur. Studies with larger sample sizes and an increased diversity of sites sampled are required to track the origin of pathogenic *Leptospira* in soil and determine relationships with environmental characteristics potentially obscured by our relatively small sample size.

### Intermediate *Leptospira* spp. are common in soil

The concentrations of *Leptospira* in soil detected using the 16S qPCR were higher than those detected by *lipL*32 in all samples. Moreover, the difference between both measurements was higher than 0.60 log_10_ units in all but one sample (mean difference and SD: 2.05±0.89 log_10_ units). Since 16S qPCR detects pathogenic and intermediate species while *lipL32* qPCR detects only pathogenic ones and both methods exclude saprophytic *Leptospira* (S2 Table), the observed concentration differences suggest that most of the signal detected with 16S qPCR originates from intermediate species. Hence, *Leptospira* species from the intermediate group may be more ubiquitous and present in significantly higher concentrations in soil from this community relative to pathogenic ones.

Intermediate species such as *L*. *fainei*, *L*. *licerasiae*, *L*. *wolffii*, and *L*. *broomii* have been linked to human leptospirosis cases [54–58], although they are not considered as virulent as the species from the pathogenic group, and thus are less relevant from a public health perspective. It is important to note that no cases of leptospirosis caused by an intermediate species has been reported in Pau da Lima during 15 years of active surveillance [6,18,30,59]. Previous studies of pathogenic *Leptospira* in the environment using 16S qPCR [20,49,60] might have led to a overestimation of the burden of the pathogen. Our results illustrate the importance of using highly specific tests to detect pathogenic bacteria in estimations of disease burden and environmental reservoir load.

### Limitations

Inherent limitations of qPCR in environmental samples may influence the accuracy of our estimates and ability to evaluate risk associations. First, qPCR may be detecting DNA from dead or damaged cells that have lost the ability to cause infection and therefore, our results may overestimate the environmental risk. On the other hand, although we optimized sample processing, DNA extraction, and detection methodologies to reduce DNA loss and PCR inhibition, we may have underestimated pathogenic *Leptospira* loads in the soil if they were below the LOD. Second, our sampling scheme may not have completely captured the heterogeneity in the urban slum environment. While we evaluated three study sites representative of different microenvironments within the slum, there may be additional heterogeneity with respect to soil type, climatic conditions, land use, and rat activity levels, which should be further explored. Finally, the relatively small sample size limited our ability to draw robust conclusions concerning environmental factors contributing to positive and negative soil samples.

### Conclusion

To date, most research regarding the environmental reservoirs of pathogenic *Leptospira* has focused on water matrices such as sewage, puddles, wells, or freshwater. Our results are the first to successfully quantify the burden of pathogenic *Leptospira* in soil from an endemic region, and indicate that soil is an additional environmental reservoir in the life cycle of pathogenic *Leptospira*. As with other environmentally transmitted diseases, the mobilization of leptospires from the sub-surface soil, either by heavy rainfall, flooding, or excavation, would contribute to environmental exposures with a sufficient dose to produce infection in humans. Furthermore, understanding the specific role and impact of soil as an environmental reservoir and the relation of low environmental concentrations to the risk of human disease is critical to our knowledge of the leptospirosis transmission cycle. Importantly, our data suggest that efforts to eliminate or reduce access to recognized transmission sources, such as open sewers, may not be sufficiently effective to decrease the risk of infection. Consequently, the role of soil in the transmission dynamics and epidemiology of leptospirosis should be considered when designing public health interventions in endemic areas.

## Supporting information

S1 Fig Precipitation during the study period at Pau da Lima community. The vertical dashed lines indicate the collection date at each sampling site.
(TIF)

S1 Supplementary methods DNA extraction efficiency from soil. (DOCX)

S1 Table Primers and probes used in this study. (DOCX)

S2 Table Results of the 16S rRNA qPCR for 21 *Leptospira* species. (DOCX)

S3 Table Highest similarity in the GenBank database of the 10 sequenced *lipL32* positive samples. (DOCX)
