# Supplementary figures and images for: Quantification of pathogenic Leptospira in the soils of a Brazilian urban slum
Source: PLoS Negl Trop Dis. 2018 Apr 6;12(4):e0006415. doi: 10.1371/journal.pntd.0006415 (PMC5906024; doi:10.1371/journal.pntd.0006415)

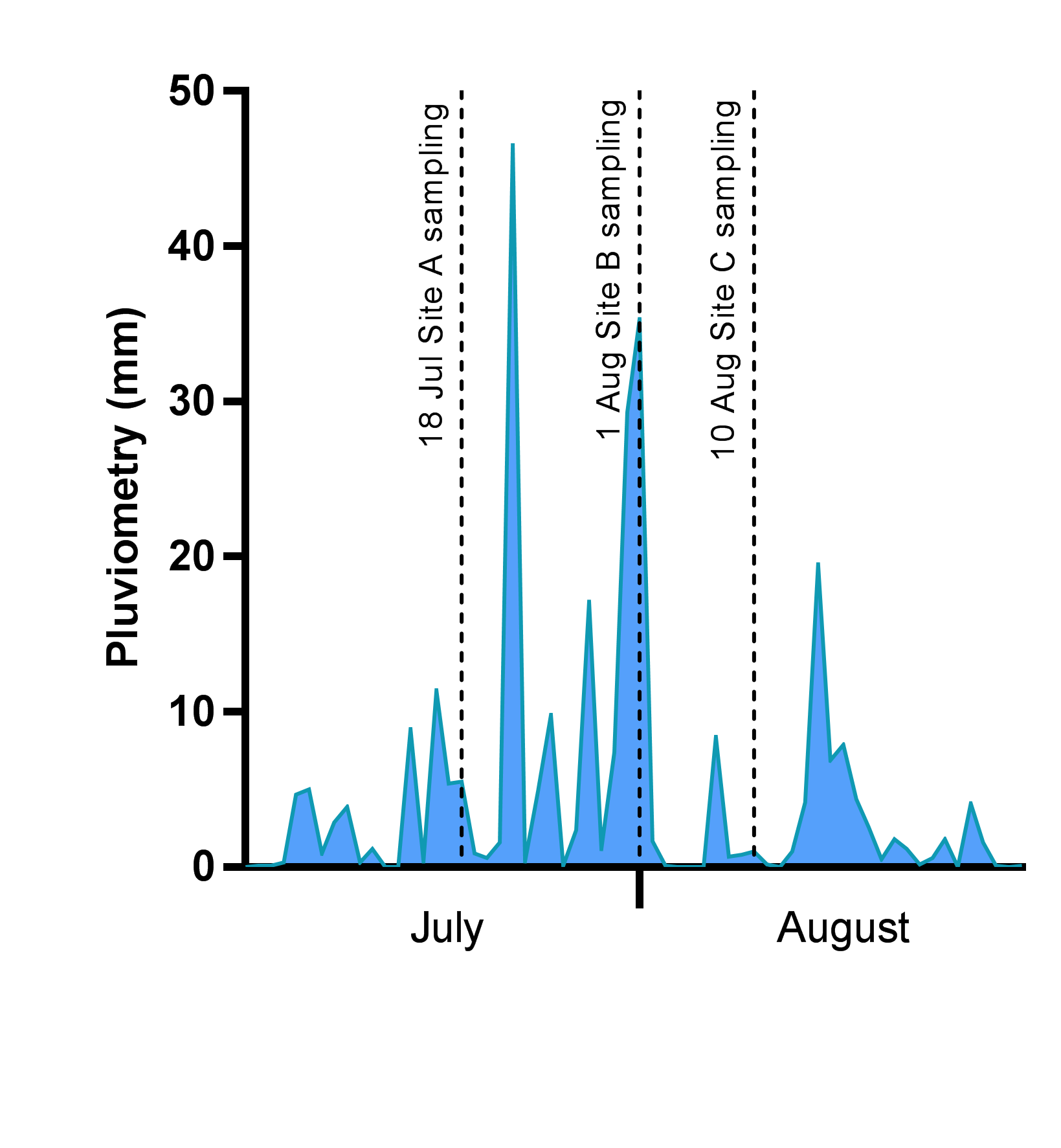

Supplement: S1 Fig — The vertical dashed lines indicate the collection date at each sampling site. (TIF) [file pntd.0006415.s001.tif]
