# Supplementary material for: Quantification of pathogenic Leptospira in the soils of a Brazilian urban slum
Source: PLoS Negl Trop Dis. 2018 Apr 6;12(4):e0006415. doi: 10.1371/journal.pntd.0006415 (PMC5906024; doi:10.1371/journal.pntd.0006415)
Supplement: S1 Supplementary methods — (DOCX) [file pntd.0006415.s002.docx]

**S1. Supplementary methods. DNA extraction efficiency from soil**

To determine the DNA extraction efficiency from soil in our site, we performed a controlled spiking experiment. Briefly, two soils were collected in sites A and C before the sampling campaign. Six subsamples of 5 g each were spiked with *L. interrogans* strain Fiocruz L1-130 at concentrations from 1 × 10^6^ cells/g to 1 × 10^1^ cells/g. Samples were mixed with 40 mL of sterile double-distilled water, vortexed at maximum speed for 2 minutes, and centrifuged at 100 x g for 5 minutes. The supernatant was recovered and centrifuged at 12,000 x g for 20 minutes at room temperature. The pellets were recovered, resuspended in 1.5 mL of sterile double-distilled water, and centrifuged at 12,000 x g for 20 minutes. Finally, the samples were decanted and the pellets were extracted using the PowerSoil DNA Isolation Kit (Mo Bio Laboratories). The spiking experiment was performed twice independently for each soil type. *Leptospira* DNA was quantified using the *lipL32* qPCR following the same protocol as the soil samples collected in the urban slum. We observed that the extraction efficiency followed a linear model (Figure S1):

Soil A: log_10_ *Leptospira* detected = 0.84 (log_10_ *Leptospira* spiked) ̶ 0.13, R^2^ = 0.98

Soil B: log_10_ *Leptospira* detected = 0.91 (log_10_ *Leptospira* spiked) ̶ 0.16, R^2^ = 0.99


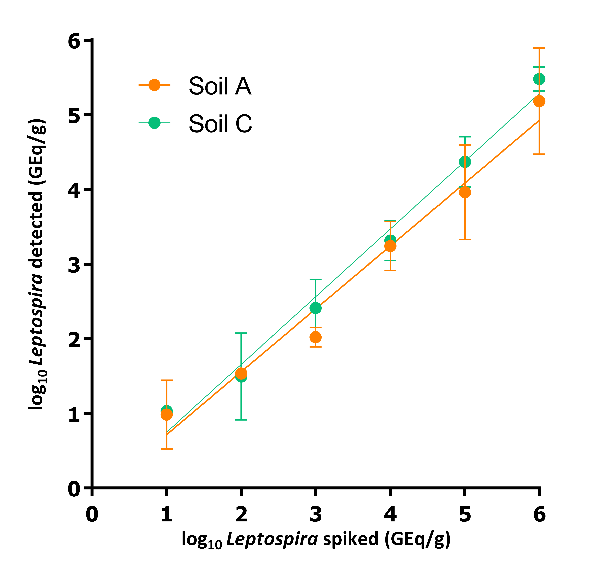


There were no significant differences between the slope or Y-intercepts of the two soils (p > 0.15). The loss of DNA varied across the range of concentrations but it was always less than 1 log_10_ units. For samples spiked with 1 × 10^1^ to 1 × 10^2^ to cells/g, losses were approximately 0.5 log_10_ units.
