# Supplementary material for: Quantification of pathogenic Leptospira in the soils of a Brazilian urban slum
Source: PLoS Negl Trop Dis. 2018 Apr 6;12(4):e0006415. doi: 10.1371/journal.pntd.0006415 (PMC5906024; doi:10.1371/journal.pntd.0006415)
Supplement: S1 Table — (DOCX) [file pntd.0006415.s003.docx]

**S1 Table.** Primers and probes used in this study.

| **Assay target** | **Primer or probe** | **Sequence (5’→ 3’)** | **Reference** |
| --- | --- | --- | --- |
| *LipL32* | *LipL32*-45F | AAG CAT TAC CGC TTG TGG TG | [1] |
|  | *LipL32*-286R | GAA CTC CCA TTT CAG CGA TT |  |
|  | *LipL32*-189P | FAM-AAA GCC AGG ACA AGC GCC G-BHQ1 |  |
| 16S RNA | Lepto F  Lepto R^a^  Probe | CCC GCG TCC GAT TAG  TCC ATT GTG GCC G**R**A CAC  FAM-CTC ACC AAG GCG ACG ATC GGT AGC-TAMRA | [2] |

**^a^** Note that primer Lepto R is degenerated at position 14 allowing for the hybridization with T and C bases.

1. Stoddard RA, Gee JE, Wilkins PP, McCaustland K, Hoffmaster AR (2009) Detection of pathogenic Leptospira spp. through TaqMan polymerase chain reaction targeting the LipL32 gene. Diagn Microbiol Infect Dis 64: 247–255.

2. Smythe L, Smith I, Smith G, Dohnt M, Symonds M, et al. (2002) A quantitative PCR (TaqMan) assay for pathogenic *Leptospira spp*. BMC Infect Dis 2: 13.
