# Supplementary material for: Quantification of pathogenic Leptospira in the soils of a Brazilian urban slum
Source: PLoS Negl Trop Dis. 2018 Apr 6;12(4):e0006415. doi: 10.1371/journal.pntd.0006415 (PMC5906024; doi:10.1371/journal.pntd.0006415)
Supplement: S2 Table — (DOCX) [file pntd.0006415.s004.docx]

**S2 Table.** Results of the 16S rRNA and *lipl32* qPCRs for 21 *Leptospira* species.

|  |  |  |  | **16S rRNA** | ***lipl32*** |
| --- | --- | --- | --- | --- | --- |
| **Group** | **Species** | **Serovar** | **Strain** | **Ct value** | **Ct value** |
| **Pathogenic** | *L. alexanderi* | Manhao 3 | L60^T^ | 14.66 | 28.86 |
|  | *L. alstonii* | Pingchang | 80-412^T^ | 15.66 | 21.35 |
|  | *L. borgpetersenii* | Hardjo-bovis | 203 | 14.91 | 15.64 |
|  | *L. interrogans* | Copenhageni | Fiocruz L1-130 | 14.59 | 15.18 |
|  | *L. kirschnerii* | Cynopteri | 3522C^T^ | 14.65 | 15.16 |
|  | *L. kmetyi* | Malaysia | Bejo-Iso9^T^ | 15.14 | 18.60 |
|  | *L. mayottensis* | **-** | 200901116 ^T^ | 14.52 | 26.67 |
|  | *L. noguchii* | Panama | CZ 214^T^ | 15.48 | 15.90 |
|  | *L. santarosai* | Shermani | 1342 K^T^ | 14.47 | 14.93 |
|  | *L. weilii* | Vughia | LT 89-68 | 15.39 | 16.08 |
| **Intermediate** | *L. broomii* | - | 5399^T^ | 15.79 | No signal |
|  | *L. fainei* | Hurtsbridge | BUT 6^T^ | 15.23 | No signal |
|  | *L. inadai* | Lyme | 10^T^ | 15.42 | No signal |
|  | *L. licerasiae* | Varillal | VAR 10^T^ | 15.46 | No signal |
|  | *L. wolffii* | - | Khorat-H2^T^ | 15.56 | No signal |
| **Saprophytic** | *L. biflexa* | Patoc | Patoc 1^T^ | No signal | No signal |
|  | *L. meyeri* | Hardjo | Went 5 | No signal | No signal |
|  | *L. terpstrae* | Hualin | LT 11-33^T^ | No signal | No signal |
|  | *L. vanthielii* | Holland | Waz Holland^T^ | No signal | No signal |
|  | *L. yanagawae* | Saopaulo | Sao Paulo^T^ | No signal | No signal |
