# Supplementary material for: Quantification of pathogenic Leptospira in the soils of a Brazilian urban slum
Source: PLoS Negl Trop Dis. 2018 Apr 6;12(4):e0006415. doi: 10.1371/journal.pntd.0006415 (PMC5906024; doi:10.1371/journal.pntd.0006415)
Supplement: S3 Table — (DOCX) [file pntd.0006415.s005.docx]

**S3 Table.** Highest similarity in the GenBank database of the 11 sequenced *lipL32* positive samples sequenced. The sequences were too short for submission to GenBank.

| **Sample** | **Site** | **Amplicon size (bp)** | **Highest similarity in GenBank** |
| --- | --- | --- | --- |
| **S16** | A | 189 | 100% *L. interrogans* strain VG99 *lipL32* gene KY356966.1 |
| **S21** | A | 141 | 96% *Leptospira sp.* strain 179507 *lipL32* gene KY356950.1 |
| **S25** | A | 172 | 93% *L. alstonii* strain GWTS #1 CP015217.1 |
| **S29** | A | 129 | 92% *L. interrogans* serovar Canicola strain 114 CP022883.1 |
| **S35** | A | 192 | 99% *L. interrogans* serovar Canicola strain 114 CP022883.1 |
| **S98** | B | 194 | 92% *Leptospira sp.* strain 179507 *lipL32* gene KY356950.1 |
| **S163** | C | 193 | 92% *Leptospira sp.* strain 179507 *lipL32* gene KY356950.1 |
| **S176** | C | 174 | 96% *L. alstonii* strain GWTS #1 CP015217.1 |
| **S179** | C | 184 | 93% *Leptospira sp.* strain 179507 *lipL32* gene KY356950.1 |
| **S183** | C | 196 | 92% *Leptospira sp*. strain 179507 *lipL32* gene KY356950.1 |
| **S186** | C | 191 | 99% *L. interrogans* serovar Canicola strain 114 CP022883.1 |
